# Haptic exploration improves performance of a laparoscopic training task

**DOI:** 10.1007/s00464-020-07898-6

**Published:** 2020-09-01

**Authors:** Roelf R. Postema, Leonie A. van Gastel, Sem F. Hardon, H. Jaap Bonjer, Tim Horeman

**Affiliations:** 1 Department of Surgery, University Medical Centers Amsterdam, Location VUMC, De Boelelaan 1117, 1081 HV Amsterdam, The Netherlands; 2 Faculty of Biomedical Engineering, University of Technology Delft, Mekelweg 2, 2628CD Delft, The Netherlands

**Keywords:** Laparoscopy training, Box trainer, Tactile exploration, Haptics, ForceSense

## Abstract

**Background:**

Laparoscopy has reduced tactile and visual feedback compared to open surgery. There is increasing evidence that visual and haptic information converge to form a more robust mental representation of an object. We investigated whether tactile exploration of an object prior to executing a laparoscopic action on it improves performance.

**Methods:**

A prospective cohort study with 20 medical students randomized in two different groups was conducted. A silicone ileocecal model, on which a laparoscopic action had to be performed, was used inside an outside a ForceSense box trainer. During the pre-test, students either did a combined manual and visual exploration or only visual exploration of the caecum model. To track performance during the trials of the study we used force, motion and time parameters as representatives of technical skills development. The final trial data were used for statistical comparison between groups.

**Results:**

All included time and motion parameters did not show any clear differences between groups. However, the force parameters Mean force non-zero (*p* = 004), Maximal force (*p* = 0.01) Maximal impulse (*p* = 0.02), Force volume (*p* = 0.02) and SD force (*p* = 0.01) showed significant lower values in favour of the tactile exploration group for the final trials.

**Conclusions:**

By adding haptic sensation to the existing visual information during training of laparoscopic tasks on life-like models, tissue manipulation skills improve during training.

**Electronic supplementary material:**

The online version of this article 10.1007/s00464-020-07898-6 contains supplementary material, which is available to authorized users.

In the training of laparoscopic surgery residents require more training compared to “open” surgery to successfully refine their sensorimotor system [1] due to the limitations of 2D vision (on the flat video display panel), difficult hand–eye coordination in the small 3D working space and the use of counterintuitive instruments with distorted tactile feedback.

Despite rapid technological advancements in the simulators including addition of force tracking and visual or haptic feedback, learning of a specific laparoscopic task is still very demanding and time-consuming. Because of work hour restrictions in the EU, UK and USA there is a pressing need for new models and techniques to make training more efficient and the learning curve for laparoscopic operations steeper [2, 3].

Our hypothesis, which has not been investigated previously, was that by allowing trainees to haptically explore an object before executing a laparoscopic action on that object, the action can be performed better and safer (i.e. with lower force parameter outcomes), can be performed quicker (i.e. shorter task time) or more efficient (less path length).

Therefore, a study was performed in which students executed basic laparoscopic tasks in a box trainer with or without a pre-test haptical exploration of the object on which they had to do the “surgical” action.

## Methods

An open randomized controlled trial. Experiment was conducted at the Amsterdam University Medical Centre, location VU Medical Centre.

**Fig. 1:**
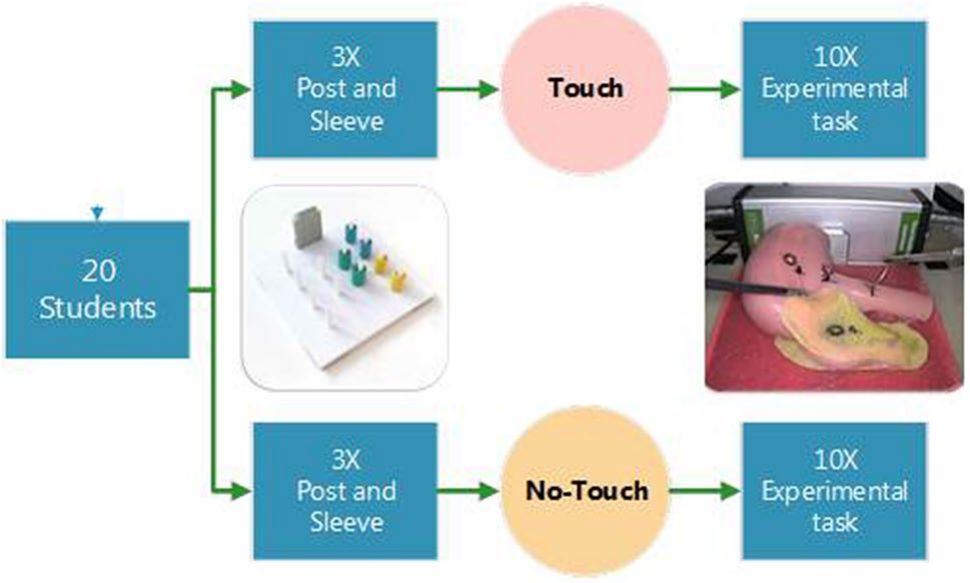
Graphical representation of the design of the study. Twenty students were divided in either a group of 10 that was allowed to touch the model or a group of 10 that was only allowed to see the model

For an overview of the study design, see Fig. 1. Participants were randomized between two groups using block randomization (with random block sizes between 4 and 8) by using www.randomization.com. This resulted in equal allocation in both groups.

### Subjects

Subjects were recruited using a social media post, posters inside the medical faculty and direct approach form researchers. To be eligible for participation they had to meet the following criteria: medical student, no visual or haptic handicaps (e.g. colour blindness, neuropathy). Additional information such as gender, age, previous laparoscopic experience, years in medical school and gaming habits was gathered from all subjects. All participants signed an informed consent form. The study was conducted in accordance with the guidelines for experimental investigation with human subjects of the University Medical Centres Amsterdam. Participation in the study did not have any consequences for their academic progress. All participants who enrolled in the study completed the experiment.

### Tasks

Task 1 (see Fig. 1), ‘Post and Sleeve’ (3Dmed, Franklin, Ohio, USA) is a previously validated laparoscopic task performed with two curved Maryland dissection forceps [4]. Task 2 (see Fig. 1 and video 1) ‘Experimental task’ was created especially for this experiment to simulate tissue manipulation. It uses a silicone ileocecal model (Simsei© Appendectomy model, Applied Medical, Rancho Santa Margarita, California, USA). Besides being very life-like in shape it was chosen especially because of its difference in the “tissue” consistency of- and between the “small bowel”, “large bowel”, “Appendix” and “meso-appendix”. For further description of the tasks we refer to Appendix.

### Study design (see Fig. 1)

**Fig. 2:**
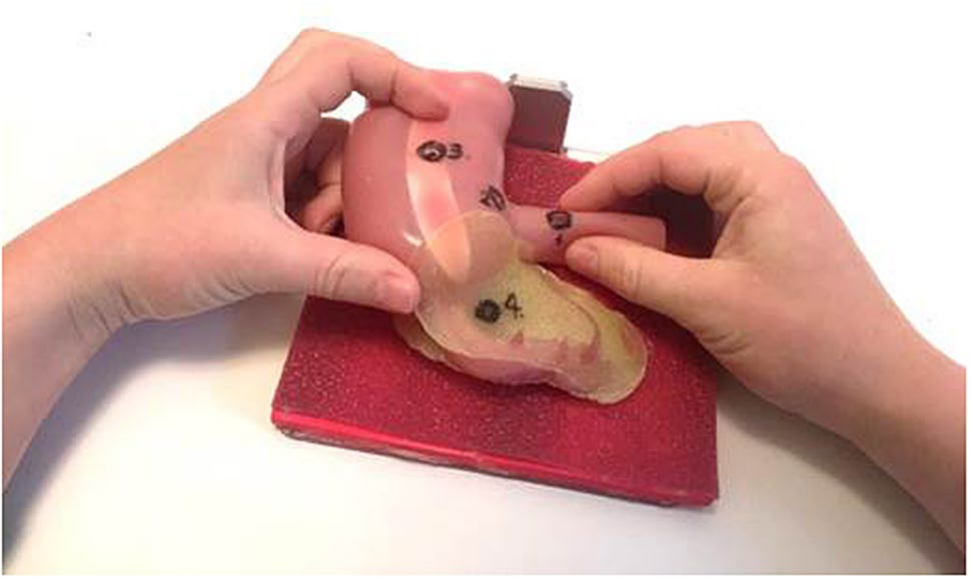
Haptic exploration of the silicone ileocecal model. The circles and numbers indicate the place and the order in which a needle should be driven through the structures

In order to get used to the equipment and compare baseline laparoscopic performance, both groups started the experiment by performing three repetitions of a standard laparoscopic “post and sleeve” task (Task 1) on a box trainer. Beforehand, they watched video-instructions on how to perform task 1. Subsequently, all participants watched the video-instructions for task 2: a task on a silicone life-like anatomic ileocecal model. Group A (Touch group) was instructed to take the anatomic model in their hands and to explore the geometry and different structures of the model with their hands (Fig. 2) for the duration of two minutes. The subjects in Group B (No-Touch group) were not allowed to touch the model but were instructed to attentively look at the model from all angles for a period of 2 min without restrictions on the distance between face and task. Thereafter, both groups performed ten repetitions of task 2. The performance of the first and the last repetition was compared between groups. The total time needed for the experiment was 40 min per participant.

### Materials and measurements

**Fig. 3:**
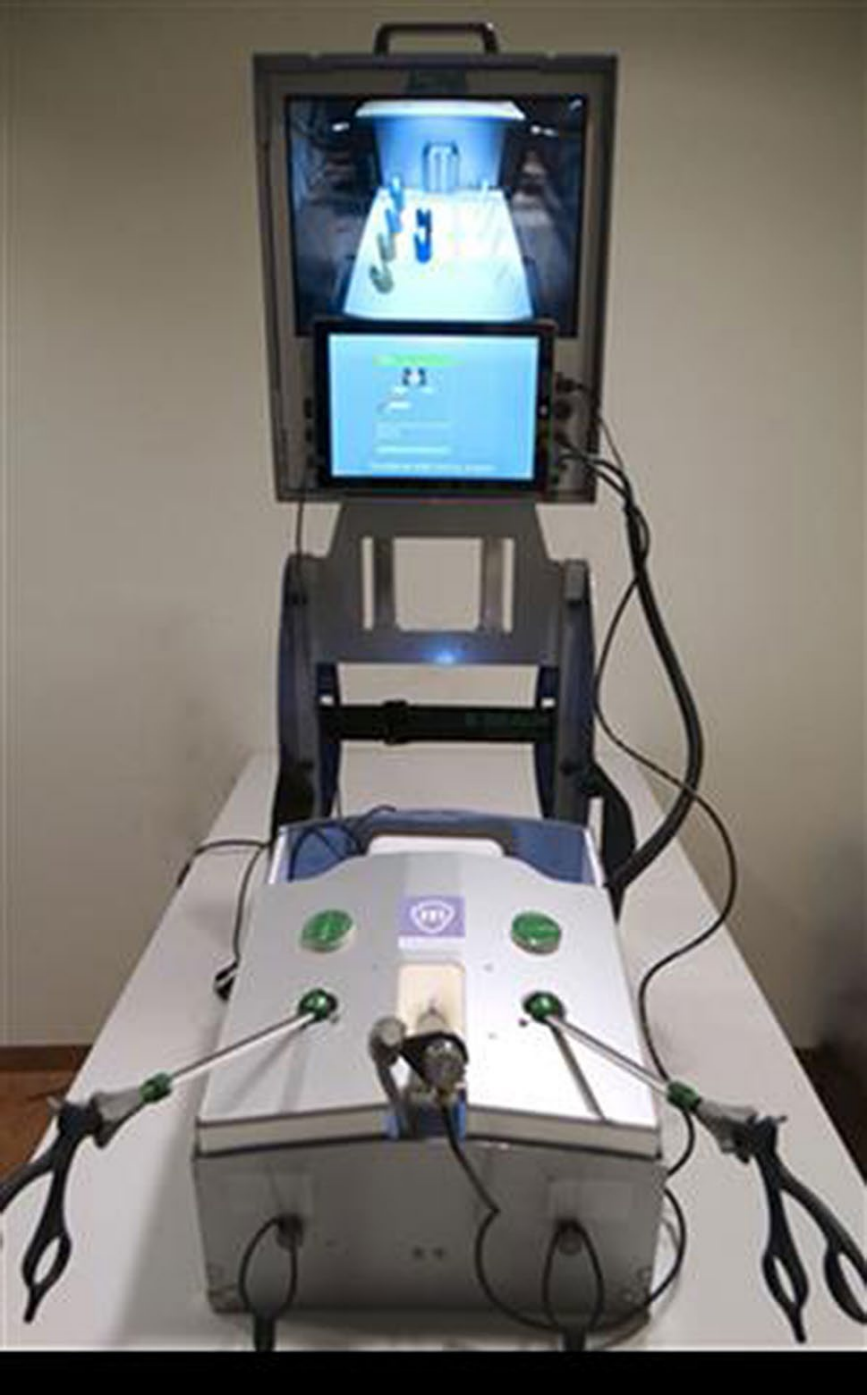
The LAPSTAR box trainer (Camtronics, Son en Breugel, The Netherlands) equipped with the ForceSense sensor and the hard- and software package that allows for tissue interaction force and 3D instrument motion measurements

Both tasks were performed on the LAPSTAR box trainer (Camtronics, Son en Breugel, The Netherlands) (Fig. 3). The box was equipped with the ForceSense measuring system (MediShield, Delft, The Netherlands). This system provides force-, motion- and time parameter outcomes used for objective assessment of laparoscopic performance [5]. It consists of a decoupled three degrees of freedom (DF) force sensor measuring force exerted on the task by the laparoscopic instruments. Furthermore, it registers three DF movements of the instruments and the time needed to complete the task. Based on their proven discrimination power, the following seven parameters were analysed: time needed to complete the task, total distance travelled by the tips of the instruments (path length), mean force, maximal force, maximal impulse, standard deviation of force and force volume. For a more detailed description of the parameters used, see Table 1. In addition, video recordings were captured of each trial.

**Table 1:** Description of parameters

Parameter	Unit	Description
Time	Seconds	Time, measured from the beginning of the task until task completion
Path length	Millimetres	Total distance travelled by the tip of the right and left instrument during the task
Mean force NZ	Newton	Mean absolute force exerted by the instruments on the task platform during periods when force is not zero
Maximal force	Newton	The highest absolute force as exerted by the instruments on the task platform during the task
Maximal impulse	Newton seconds	The largest product of force and the duration that the force was exerted, before force returned to zero (the area under a force peak if force is presented in time)
Force volume	Cubic Newton (N^3^)	The volume of an ellipsoid fitted around the standard deviations of the force along three principal components. (High force volume indicates fast increasing and decreasing forces in different directions.)
SD Force	Newton	The standard deviation of the absolute force

*NZ* non-zero, *SD* standard deviation

### Analysis

For data analysis, IBM SPSS Statistics version 22.0 was used with non-parametric tests as the data were not normally distributed. To compare baseline characteristics between group A and B in the pre-test on task 1, the non-parametric Mann–Whitney *U* test was used. The same test was used to compare the different performance parameter outcomes for task 2 between groups in the final trials. Differences were considered statistically significant if *p* < 0.05 (tested double-sided).

## Results

### Participants

As all subjects were able to finalize the different elements of the experiment in time, a total of 20 subjects were included in the analysis. Baseline characteristics are shown in Table 2. The two groups were comparable since no significant differences were found in age, sex or gaming experience. In addition, all participants did have little to no laparoscopic experience on a training system and no experience in laparoscopic surgery.

**Table 2:** Baseline characteristics

Baseline characteristics	Touch	No touch	*p*-value
Age^a^	22.5 (18–25)	21.5 (19–24)	0.236
Sex	M 20%, F 80%	M 20%, F 80%	1.0
Medicine year^a^	4.5 (2–6)	4.0 (2–6)	0.536
Lap. experience simulator (min^a^)	0 (0–90)	0 (0–5)	0.101
Lap experience OR assisting (times^a^)	0 (0–2)	0 (0–3)	0.426
Gaming (h/week^a^)	0 (0–28)	0 (0–28)	1.0

Mann–Whitney *U* test

^a^Median (range)

### Baseline laparoscopic performance

Baseline laparoscopic performance of the participants was assessed using task 1, ‘Post and sleeve’. During the first two trials, participants familiarized themselves with the laparoscopic equipment. Analysis of the third trial showed no significant differences in time, motion or force parameters (Table 3).

**Table 3:** Baseline performance (Task 1: peg transfer)

Baseline laparoscopic performance	Touch	No touch	*p*-value
Time (s)	156.51	169.32	0.406
Path length (mm)	7589.10	8916.98	0.257
Mean force non-zero	0.42	0.48	0.096
Maximal force (N)	1.53	2.41	0.140
Maximal impulse (Ns)	2.90	3.30	0.226
Force volume (N^3^)	0.04	0.07	0.088
SD force (N)	0.18	0.25	0.053

Medians, MWU test

### Performance on experimental task

**Fig. 4:**
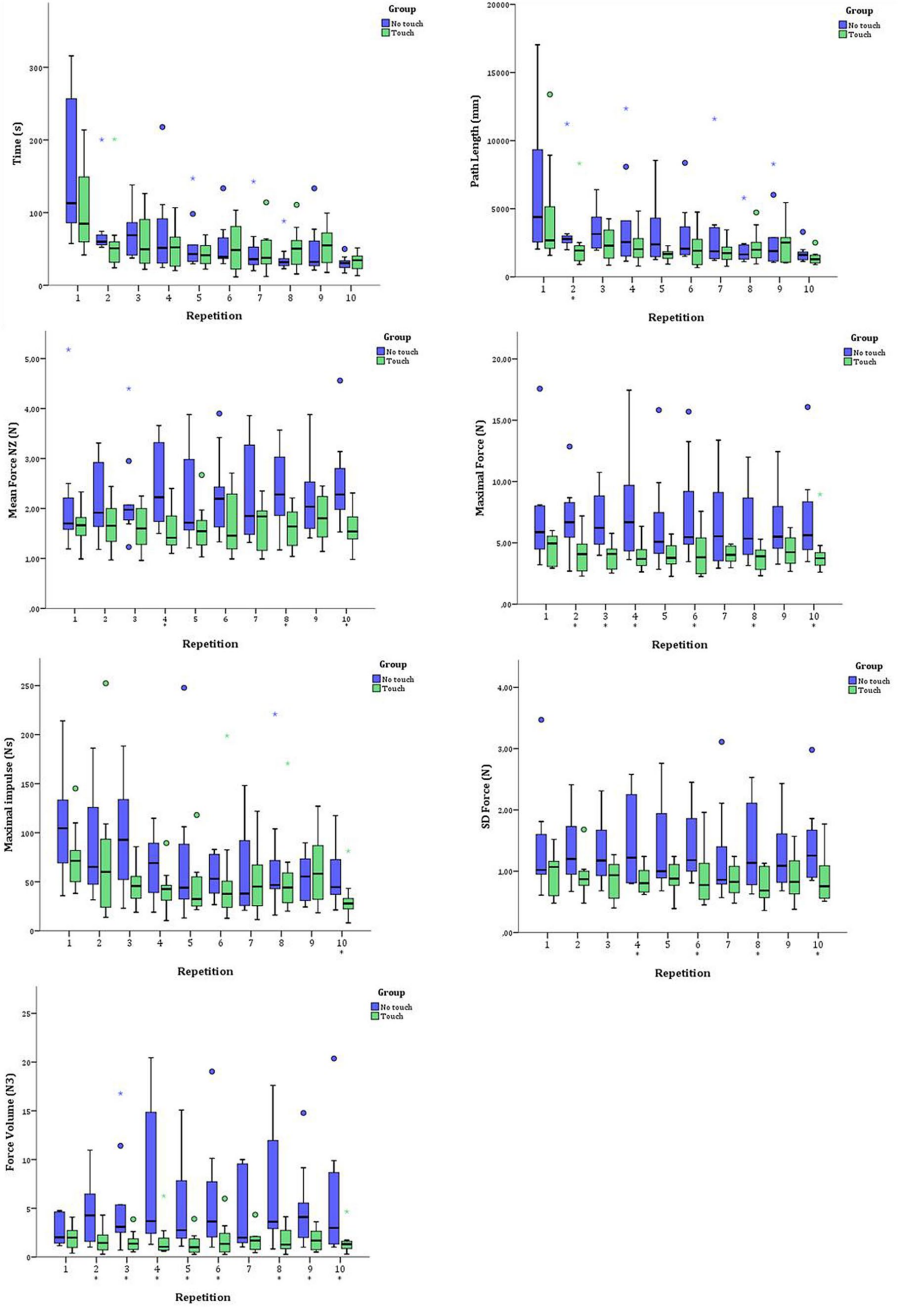
Graphic representation of median and standard deviation outcomes of different parameters for the 10 repetitions of the experimental task 2. In blue the no-touch group and in green the touch group

Figure 4 shows boxplots of the seven different performance parameters for each repetition. One can see from these figures that there is no difference for time and path length for both groups. This was statistically confirmed although for path length a trend seems to be present towards less path length used in the “Touch” group. Looking at all four force parameters, values are lower and ranges are smaller in the Touch group. Statistical analysis values show a significant reduction for all force parameters (Table 4).

**Table 4:** Performance experimental task (Task 2)

Performance task 2	Touch	No touch	*p*-value
Time (s)	34.43	30.55	0.496
Path length (mm)	1294.13	1613.67	0.096
Mean force non-zero	1.54	2.28	**0.004**
Maximal force (N)	3.75	5.62	**0.010**
Maximal impulse (Ns)	27.90	44.52	**0.019**
Force volume (N^3^)	1.32	2.99	**0.023**
SD force (N)	0.76	1.26	**0.013**

Medians, MWU test

Bold values are statistically significant for *p*-value

## Discussion

Similar as in airline and air force pilot training, a number of surgical simulators have been designed and validated for laparoscopic skills training. Psychomotor skill learning can be achieved with simulation, reaching learning curves in a more expedite fashion [6]. Simulation training has also been proven to decrease error rates and improve patient care and performance in the OR [7–9].

Alternative mental training techniques have been investigated as an adjunct to physical surgical skills training (e.g. systematically and repeatedly imagining an object movement without actually performing it) but so far, for surgical training, the results have been variable [10, 11].

In learning a surgical technical task it seems clinically very relevant that not only the interaction between hands and laparoscopic instruments is important but also the effect this interaction has on the object on which it is performed. Good and solid neurosensory information about the object could therefore be advantageous in order to avoid tissue damage or blood flow reduction. Everyday visual object recognition is not a passive task but involves active exploration using other organs, principally the hands. Besides the help they provide for visual recognition (by manipulating the object it can be viewed from more angles) the hands, through their ability to feel shape, texture, consistency, vibration, temperature and other tissue properties provide tactile sensory information. Together with kinaesthetic perception from receptors in muscles, tendons and joints which provide position, movement and force information this leads to the haptic perception in our brain [12].There is increasing evidence in the neuropsychological literature that visual and haptic information combine or converge to form a mental representation of an object which, because of the combination of these inputs, is more refined and robust than the representation formed by only one of these sensory inputs [13–16]. Haptic information is defined as the combination of sensory input through the tactile receptors in the skin and the kinesthesic receptors in muscles, tendons and joints. Its value is paramount in open surgery, where “feeling” the tissues is as important as visualizing them [17]. Only a few studies have been conducted comparing haptic exploration versus exploration with instruments on an object in a minimally invasive surgery setting, but the results are invariably in favour of the haptic exploration group [18, 19].

Our novel hypothesis was that by allowing trainees to physically explore an object before executing a laparoscopic action on that object, the action can be performed safer (i.e. lower force parameter outcomes), more efficient (less path length) and can be performed more quickly (i.e. shorter task time). The results indicated that this hypothesis holds for the force parameters that represent safe tissue-handling. We postulated that the “old fashioned” haptic interaction with the patients’ living and healthy, diseased or altered tissue contributes to the “mental image” and “memory” of the tissue, organ or body part that surgeons develop. Especially of how to handle, interpret or predict the reaction of a tissue, organ or body part while manipulating it during an operation. Our findings suggest that it seems likely that in training residents for a particular (laparoscopic) skill, attentive haptic exploration of the object on which the skill has to be learned, could lead to better performance of the skill. Conducting an experiment in a “top down” approach using fMRI imaging or extensive neuropsychological testing seemed too complicated to attain relevant results with the relatively small experimental groups we had at our disposal. Therefore we chose the more surgical “bottom up” approach where we compared the visual only- to the visual combined with haptic exploration of an experimental skill training object. Our silicone model was, by expert surgeons, experienced as very life-like in shape but especially in its difference in the “tissue” consistency between the “small bowel”, “large bowel”, appendix” and “meso-appendix”. Because of this haptic difference and the clinically relevant shape it proved to be a good model to test the effect of haptic exploration.

The most striking finding in our study is that significant less force is used in the group who had been able to haptically explore the experimental object before performing the action on that object. This can be seen in all force measurements as indicated in Fig. 3 and Table 4.

The use of less force has been previously validated as being a good indicator of proper, more respectful and safer tissue-handling [20, 21]. The use of less force is, in other studies, also a recurring indicator of more experience in performing the action or more experience with tissue and instrument handling as seen in experts compared to novices [22]. One can interpret this as that the action is performed more subtle and with more “feeling”, indicating better anticipation to the tissue properties. Other studies show that the same kind of difference in force reduction is seen when haptic feedback is added to instruments in a virtual reality laparoscopic skill training action [22]. So, the reduction in force measured in our study in the “haptic” group can be interpreted either as an indicator of more sensitivity (or a lower threshold) to haptic feedback through the laparoscopic instruments and/or of an already existing experience with the tissue properties stored in the memory of our subjects as a result of the haptic exploration of the object.

The results from our study show that the amount of time or amount of movement, as indicated by total path length travelled by the instruments, was not significantly different, although there is an indication of a trend towards less path length as can be seen in Fig. 3. A post-hoc power analysis (samplesizepwr.m, Mathworks, Natick, MA), based on the task time and path length data indicates a trend towards a significant reduction of path length and Task time if 111 and 749 participants are included. There are however studies that show that the amount of time for an action to be completed is not an adequate indicator of technical competence in performing or learning a task for novices [23]. So the lack of significant difference between our groups for these parameter outcomes does seem less relevant.

The outcomes of this study are relevant in two ways. First it shows that surgical trainees can profit from haptic exploration of 3D models of complex organs before surgery and secondary it indicates the need for more sensitive instruments that allow for accurate haptic feedback.

More research is needed to clarify the exact neuropsychological explanation of our positive results but the effect is significant. Therefore haptic exploration of an object before doing a laparoscopic action on that object seems to be a relevant addition to laparoscopic skills training.

Limitations of this study are that it was performed using medical students and not surgical residents, which, we think, could have led to other and probably even more accentuated differences. Further studies should include an inventory of hobbies/sports that potentially influenced the tactile haptic maturity of an individual. What could also be of interest is to see the progression of trainees that start with different parameter levels, so a study could be conducted that includes correlations between pre and post measurements of individual trainees. Also the use of non-viable tissue models could be of limitation because of the more crude differences in the silicone models compared to human organs. And for clinical implications, the differences in haptic perception of an organ between human individuals, because of differences in sex, build, amount of fat and age, but also whether an organ is inflamed or contains malignancy can severely alter the way it “feels”, so a haptic pre-operative exploration on a “standard” ex-vitro model could limit the clinical results which is, of course also the disadvantage of training on “no touch” laparoscopic models.

In the near future we will conduct new experiments with larger group sizes based on our findings in this study and try to further prove our hypothesis.

## Conclusion

The results of this study suggest that trainees can benefit from haptic exploration on a representative model before conducting a laparoscopic action. The object is treated more gently as a result of the haptic exploration and it can therefore be a relevant addition to laparoscopic skills training.

### Electronic supplementary material

Below is the link to the electronic supplementary material.

Electronic supplementary material 1 (MP4 430 kb)

## Appendix

### Description of Tasks

#### Task 1

The task board consists of 12 posts with six sleeves around the posts on the left. The sleeves have to be picked up with the left instrument on the left side of the board, and put down with the right instrument on the opposite side. Afterwards, this is repeated in the opposite direction.

#### Task 2

The silicone ileocecal model (Simsei© Appendectomy model, Applied Medical, Rancho Santa Margarita, California, USA) has been modified to fit into the box trainer and 4 numbered holes have been added for needle driving (Fig. 2). The task is performed with a laparoscopic 5 mm needle driver (Karl Storz, Tuttlingen, Germany) in the right trocar and a curved Maryland forceps (Applied medical, Rancho Santa Margarita, California USA) in the left trocar. A blunt customized curved needle (L:30 mm; R: 20 mm) was fixed in the beak of the needle holder in a standardized way by the experimenters before each trial. Participants were instructed to insert and pass the holes in the model with the needle in a fixed order. The verbal instruction was: firstly, enter hole number 1 with the tip of the needle and exit through hole number 2. As soon as the tip of the needle is visible in hole number 2, draw back the needle and enter again through hole number 2, then showing the tip in hole number 3. Lastly, enter through hole number 4 (in the meso-appendix) and show the tip of the needle underneath the base of the appendix.

After this last step the subjects were asked to retract the instruments and needle away from the task to prevent undesired interactions that influence measurements.
